# Predictors and mortality of patients with delayed pulmonary embolism diagnosis: A cohort study

**DOI:** 10.22088/cjim.13.4.757

**Published:** 2022

**Authors:** Hassan Aghajani, Susan Hashemi, Amirali Karimi, Somayeh Yadangi, Arash Jalali, Yaser Jenab

**Affiliations:** 1 Department of Cardiology, Tehran Heart Center, Tehran University of Medical Sciences, Tehran, Iran; 2 Tehran Heart Center, Tehran University of Medical Sciences, Tehran, Iran; 3 School of Medicine, Tehran University of Medical Sciences, Tehran, Iran; 4 Cardiovascular Diseases Research Institute, Tehran Heart Center, Tehran University of Medical Sciences, Tehran, Iran

**Keywords:** Delayed diagnosis, Mortality, Pulmonary embolism, Venous thromboembolism

## Abstract

**Background:**

Some earlier studies demonstrated an increased mortality risk attributed to delayed pulmonary embolism (PE) diagnosis. Therefore, we mainly aimed to determine the predictors of diagnostic delays and the effect of delayed diagnosis on mortality.

**Methods:**

We prospectively studied 756 consecutive patients admitted with PE between March 2007 and September 2017. The delayed diagnosis was defined as (1) patient presenting > 7 days after onset of symptoms, (2) diagnosis takes > 24 hours upon arriving in the ED, or (3) undergoing coronary angiography before establishing PE diagnosis.

**Results:**

A total of 127 (16.7%) patients met the delayed group’s criteria. Heart failure (OR= 2.257, 95% CI: 1.130-4.508, P= 0.021), diabetes mellitus (OR= 1.568, 95% CI: 0.996-2.469, P= 0.052), and precordial T wave inversions (OR=2.559, 95% CI: 1.649-3.970, P< 0.001) were linked to higher rates of delayed diagnosis, while hemoptysis (OR=0.254, 95% CI: 0.059-1.087, P= 0.065) and hemodynamic instability (OR= 0.434, 95% CI: 0.168-1.123, P= 0.085) negatively correlated with it. Delayed PE diagnosis did not significantly impact the overall survival during the follow-up. The unadjusted and adjusted mortality hazard ratio for delayed diagnosis were 1.198 (95% CI: 0.758- 1.894, P= 0.439) and 1.215 (95% CI: 0.762- 1.939, P=0.413), respectively. Older age, heart failure, and hemodynamic instability increased the risk of death (p<0.001).

**Conclusion:**

Hemoptysis, hemodynamic instability, diabetes mellitus, heart failure, and T wave inversions in precordial leads were the independent predictors of delayed diagnosis. Delayed PE diagnosis did not increase the patients' mortality rates.

Pulmonary embolism (PE) is a potentially lethal condition characterized by an occlusion in the pulmonary arteries (1). Various studies estimate an annual incidence of 60-70 or 39-115 per 100,000 population (2, 3), with an increased risk in older ages 2-5. PE accounts for up to 300,000 mortalities per year in the United States, placing it among the highest cardiovascular causes of death (2, 3). Nevertheless, public awareness of this disease stood at substantially lower rates compared to ischemic heart disease and stroke (2). Owing to the acute and life-threatening nature of an episode, diagnosing this condition requires the utmost attention to alter its potentially devastating trajectory. Circulatory failure can render the patients susceptible to worse outcomes compared with the patients without hemodynamic instability and right ventricular failure (3, 4). The role of earlier diagnosis on the patient’s outcome remains a matter of debate. While some studies suggest that earlier diagnosis reduces the associated adverse outcomes (4-6), other studies reported similar results between patients identified early or late (7-9). The low specificity and sensitivity of PE signs and symptoms remain a challenge for its earlier diagnosis (5, 10).

Therefore, as high as 50% of the patients with PE might face seven days or more lag between the onset of their symptoms and diagnosis, marking it as one of the most missed conditions in the routine clinical practice (5, 10). Several risk-factors were attributed to an increased rate of delayed PE diagnosis (5, 7, 11, 12). However, the discrepancies among these variables halted the emergence of a clear prognostic picture. In this study, we mainly aimed to determine the predictors of diagnostic delays and the effect of delayed diagnosis on mortality.

## Methods

**Study design: **We studied 756 consecutive patients admitted with PE between March 2007 and September 2017 to Tehran Heart Center. The study was approved by the local institutional review board with the ethics code of IR.TUMS.MEDICINE.REC.1398.022. We did not include the patients who developed PE during their hospitalization in our center. Contrast-enhanced pulmonary multi-detector computed tomography (MDCT) angiography or ventilation-perfusion lung scintigraphy (V/Q scan) objectively confirmed the PE diagnosis. We assigned the patients into two groups of delayed and non-delayed diagnosis. The delayed diagnosis was defined as (1) patient presenting > 7 days after onset of symptoms in those not admitted through emergency department (ED), (2) diagnosis takes > 24 hours upon arriving in the ED, or (3) undergoing coronary angiography before establishing PE diagnosis 7. Patients developing cardiac arrest or hemodynamic instability were included in the delayed diagnosis group if post-mortem PE diagnosis was established.

The extracted data include demographics, past medical history, comorbidities, known PE risk factors, and patient's symptoms and physical examination findings at the time of arrival at the ED. Our central lab facilities carried out all the laboratory measurements in this study.

D-dimer levels were not requested in the first 24 hours in any of the patients in the delayed diagnosis group. We defined hemodynamic instability as a systolic blood pressure of less than 100 mmHg or the need for inotrope injection. This study was approved by the local institutional review board.

**Statistical analysis: **Continuous variables including age, heart rate, and systolic blood pressure were reported as mean with standard deviation (SD). They were normally distributed and was assessed using histogram chart and descriptive measures. Categorical variables were reported as frequency with percentage. The univariate effect of covariates on delayed diagnosis was calculated using logistic regression model. All variables with p-values< 0.15 in the univariate analysis were candidates to enter the multivariable model. A multivariable logistic regression model with backward elimination technique (considering removal and entry p-values as 0.1 and 0.05, respectively). Univariate and multivariable effects were reported through odds ratio (OR) with 95% confidence interval (CI). Calibration of the final predictive model was evaluated applying the Hosmer-Lemeshow test. The discrimination power of the final model was assessed using area under the ROC (receiver operating characteristic) curve (AUC) with 95% CI. The adjusted and unadjusted effect of delayed diagnosis on all-cause mortality was computed applying Cox’s proportional hazard model and the effects were reported as hazard ratio (HR) with 95% CI. Variables which were simultaneously associated with all-cause mortality and delayed diagnosis with p0.1 were considered as potential confounders. All statistical analyses were conducted using IBM SPSS for Windows Version 22.0 (Armonk, NY: IBM Corp.). 

## Results

**Patient characteristics: **In the study period, our center admitted 756 patients with a confirmed PE diagnosis. Of these patients, 127 (16.7%) met the delayed diagnosis group criteria, 24 (18.9%) of them undergoing coronary angiography before the PE diagnosis was established. The mean age of the included patients was 69.8±16.8) and 352 (46.4%) were females (table 1). The mean heart rate and systolic blood pressure of the patients were 100.9 and 129.9, respectively. The most prevalent symptoms were acute onset dyspnea (88.7%), pleuritic (30.3%), and non-pleuritic (19.9%) chest pains. Hypertension (41.9%), obesity (33.2%), recent immobilization (28.1%), smoking history (21.3%), coronary artery disease (19.5%), and diabetes mellitus (19.5%) were the most common underlying conditions or PE risk factors. S1Q3T3 accounted for the most common electrocardiographic pattern seen in 34.8% of the patients.

**Predictors of delay**

**- Univariate analysis: **Older age, hypertension, diabetes mellitus, heart failure, S1Q3T3 pattern, inverted T waves in precordial leads, S1S2S3 pattern, and right ventricular (RV) strain in MDCT angiography were associated with a higher risk of delayed diagnosis. On the other hand, hemodynamic instability, hemoptysis, and normal ECG were less prevalent in the delayed diagnosis group.

**Table 1:** Characteristics of the patients and their univariate effect on the delayed diagnosis

**Parameters**	**Total (N=756)**	Non-delayed Dx* (n=629)	**Delayed Dx (n=127)**	**OR (95% CI)**	**Pvalue¶**
Patient characteristics					
Age	60.4 (16.8)	59.8 (16.9)	63.0 (16.0)	1.011 (1.000-1.023)	0.057
Gender (male)	407 (53.6%)	332 (52.5%)	75 (59.1%)	1.303 (0.885-1.918)	0.179
Heart rate	100.9(19.86)	100.5 (19.59)	102.6 (21.16)	1.005 (0.996-1.015)	0.279
Systolic blood pressure	129.9 (21.62)	129.4 (21.57)	132 (21.86)	1.005 (0.997-1.014)	0.217
Presenting signs and symptoms					
Dyspnea	673 (88.7%)	561 (88.8%)	112 (88.2%)	0.945 (0.522-1.709)	0.852
Pleuritic chest pain	230 (30.3%)	190 (30.1%)	40 (31.5%)	1.094 (0.706-1.694)	0.687
Non-pleuritic chest pain	151 (19.9%)	125 (19.8%)	26 (20.5%)	1.081 (0.653-1.788)	0.762
Syncope	75 (9.9%)	63 (10.0%)	12 (9.4%)	0.942 (0.492-1.804)	0.858
Hemodynamic instability	62 (8.2%)	57 (9.0%)	5 (3.9%)	0.413 (0.162-1.053)	0.064
Hemoptysis	36 (4.7%)	34 (5.4%)	2 (1.6%)	0.281 (0.067-1.187)	0.084
Underlying conditions and PE risk factors					
Hypertension	318 (41.9%)	257 (40.7%)	61 (48.0%)	1.349 (0.920-1.977)	0.125
Obesity	252 (33.2%)	212 (33.5%)	40 (31.5%)	0.911 (0.605-1.371)	0.655
Recent Immobilization	213 (28.1%)	182 (28.8%)	31 (24.4%)	0.798 (0.514-1.240)	0.316
Smoking history	162 (21.3%)	134 (21.2%)	28 (22.0%)	1.051 (0.663-1.666)	0.832
Coronary artery disease	148 (19.5%)	125 (19.8%)	23 (18.1%)	0.897 (0.548-1.467)	0.665
Diabetes Mellitus	148 (19.5%)	114 (18.0%)	34 (26.8%)	1.661 (1.068-2.585)	0.024
Recent major surgery (<3Weeks)	108 (14.2%)	91 (14.4%)	17 (13.4%)	0.919 (0.526-1.604)	0.766
Previous DVT	65 (8.6%)	56 (8.9%)	9 (7.1%)	0.785 (0.378-1.630)	0.515
Long haul travel	64 (8.4%)	51 (8.1%)	13 (10.2%)	1.299 (0.684-2.467)	0.424
Heart failure	52 (6.9%)	39 (6.2%)	13 (10.2%)	1.734 (0.897-3.351)	0.102
Estrogen use	47 (6.2%)	38 (6%)	9 (7.1%)	1.192 (0.562-2.531)	0.647
Cerebrovascular accident	43 (5.7%)	37 (5.9%)	6 (4.7%)	0.797 (0.329-1.931)	0.616
History of malignancy	43 (5.7%)	33 (5.2%)	10 (7.9%)	1.551 (0.744-3.235)	0.241
Peripheral artery disease	5 (0.7%)	5 (0.8%)	0 (0%)	2.246 (0.123-40.869)	0.585
Electrocardiographic (ECG) findings					
Normal ECG	65 (8.6%)	59 (9.3%)	6 (4.7%)	0.482 (0.203-1.141)	0.097
S1Q3T3	264 (34.8%)	211 (33.4%)	53 (41.7%)	1.429 (0.968-2.110)	0.072
T wave inversions in precordial leads	144 (19.0%)	103 (16.3%)	41 (32.3%)	2.449 (1.596-3.755)	< 0.001
RBBB	110 (14.5%)	88 (13.9%)	22 (17.3%)	1.295 (0.776-2.161)	0.322
S1S2S3	101 (13.3%)	64 (10.1%)	37 (29.1%)	3.649 (2.299-5.790)	< 0.001
Right axis deviation	74 (9.7%)	63 (10.0%)	11 (8.7%)	0.856 (0.438-1.675)	0.651
Pulmonary MDCT angiography findings					
Segmental involvement	157 (20.7%)	127 (20.1%)	30 (23.6%)	0.820 (0.520-1.287)	0.385
Saddle emboli	127 (16.7%)	109 (17.2%)	18 (14.2%)	0.699 (0.369-1.323)	0.271
RV strain	126 (16.6%)	86 (13.6%)	40 (31.5%)	2.919 (1.884-4.524)	< 0.001

*Abbreviations: Dx: Diagnosis, OR: Odd ratio, CI: Confidence interval, PE: Pulmonary embolism, DVT: Deep vein thrombosis, RBBB: Right bundle branch block, MDCT: Multi-detector angiography, RV: Right ventricular, GI: Gastrointestinal

**Categorical variables are presented as number and percentage, while continuous variables are presented as mean (± SD)

¶ Significant variables on the univariate analysis (P< 0.15) are illustrated in bold style.

**- Multiple predictors of delayed diagnosis: **Diabetes mellitus (OR= 1.568, 95% CI: 0.996- 2.469, P= 0.052), heart failure (OR= 2.257, 95% CI: 1.130- 4.508, P= 0.021), and inverted T waves (OR= 2.559, 95% CI: 1.649- 3.970, p<0.001) in the precordial leads independently predicted an increased risk of delayed diagnosis. Hemoptysis (OR= 0.254, 95% CI: 0.059- 1.087, P= 0.065) and hemodynamic instability (OR= 0.434, 95% CI: 0.168- 1.123, P=0.085) inversely correlated with a risk of delayed diagnosis. The AUC mounted to 0.645 (table 2).

**PE treatment: **Upon admission, a total of 65 (8.2%) and 126 (16.7%) patients had hemodynamic instability and saddle emboli, respectively. Thrombolytic therapy was initiated in 111 (14.7%) patients, 103 (16.6%) of them in the non-delayed diagnosis group. Only eight (6.3%) patients in the delayed diagnosis group received thrombolytic therapy (P= 0.004) (table 3). 

Two percent of the patients underwent surgical thrombectomy, all of whom were among non-delayed diagnosis patients. Six hundred and twenty one (98.7%) of the patients in the non-delayed diagnosis group and 124 (97.6%) of the patients in the delayed diagnosis group received anticoagulant therapy (P= 0.357).

**Table 2:** Multiple predictors of delayed diagnosis

Parameters	OR*	Pvalue¶
**Diabetes Mellitus**	1.568 (0.996-2.469)	0.052
**Heart failure**	2.257 (1.130-4.508)	0.021
**Hemodynamic instability**	0.434 (0.168-1.123)	0.085
**Hemoptysis**	0.254 (0.059-1.987)	0.065
**T wave inversions in precordial leads**	2.559 (1.649-3.970)	< 0.001
**Hosmer-Lemeshow goodness of fit test: P= 0.855**		
**AUC: 64.5% (95% CI: 59.2- 69.7%)**		

*Abbreviations: OR: Odd ratio, CI: Confidence interval, AUC: Area under the curve

Enlisted variables are the significant ones in the multivariate analysis (p< 0.1)

**Table 3:** PE treatments and their major complications among groups

Parameters	Total (N= 756)	Non-delayed Dx* (n= 629)	Delayed Dx (n= 127)	P-value¶
**Anticoagulant therapy**	745 (98.5%)	621 (98.7%)	124 (97.6%)	0.357
**Thrombolytic therapy**	113 (14.9%)	105 (16.6%)	8 (6.3%)	0.004
**GI bleeding**	12 (1.6%)	8 (1.3%)	4 (3.1%)	0.133
**Heparin-induced thrombocytopenia**	10 (1.3%)	10 (1.6%)	0 (0%)	0.313
**Intracranial hemorrhage**	4 (0.5%)	3 (0.5%)	1 (0.8%)	0.660

*Abbreviations: Dx: Diagnosis, OR: Odd ratio, CI: Confidence interval significant variables (P< 0.05) are illustrated in bold style.

**Figure 1:**
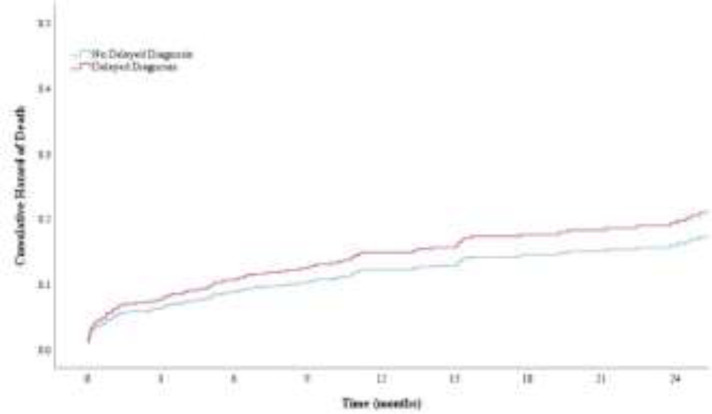
The cumulative hazard of all-cause death

**Mortality risk: **The in-hospital mortality rate was 6.7% (n=51). All-cause mortality was 19.6% (n=148) with a median follow-up time of 16.9 (95% CI: 14.8-18.9) months. The delayed diagnosis did not significantly impact the overall survival during the follow-up. The unadjusted and adjusted effect of delayed diagnosis on mortality were 1.198 (95% CI: 0.758-1.894, P=0.439) and 1.215 (95% CI:0.762- 1.939, P=0.413), respectively. Older age, heart failure, and hemodynamic instability increased the risk of death (p<0.001) (table 4). Figure 1 illustrates the cumulative hazard of all-cause mortality among delayed and not delayed diagnosis groups.

**Follow-up echocardiography data: **The follow-up echocardiography was performed about three months after admission in 610 (80.7%) patients. Other patients were dead or lost to follow-up. Patients in the delayed diagnosis group had similar rates of moderate to severe RV function (10.3% vs. 15.1%, P=0.200) and a higher rate of enlarged RV diameter (> 35 mm) (23.4% vs. 13.9%, P= 0.015) (table 5). 

**Table 4:** The effect of delayed diagnosis on the all-cause mortality

Parameters	HR* (95% CI)	Pvalue¶
**Unadjusted **	1.198 (0.758-1.894)	0.439
**Adjusted**	1.215 (0.752-1.939)	0.413
**Age**	1.028 (1.016-1.041)	< 0.001
**Gender**	0.884 (0.631-1.238)	0.474
**Hypertension**	0.990 (0.691-1.418)	0.956
**Diabetes mellitus**	1.025 (0.676-1.552)	0.908
**Heart failure**	2.243 (1.443-3.486)	< 0.001
**Hemodynamic instability**	4.787 (3.264-7.022)	< 0.001

*Abbreviations: HR: Hazard ratio, CI: Confidence interval

¶ Significant variables (P< 0.05) are illustrated in bold style

**Table 5:** Follow-up echocardiography results

Echocardiographic parameters	Non-delayed Dx* (n= 503) No (%)	Delayed Dx (n= 107) No (%)	Pvalue¶
**Moderate to severe RV dysfunction**	76 (15.1)	11 (10.3)	0.200
**RV diameter > 35 mm**	70/502 (13.9)	25 (23.4)	0.015

*Abbreviations: Dx: Diagnosis, RV: Right ventricular

¶ Significant variables (p< 0.05) are illustrated in bold style

## Discussion

Previous studies reported a clinical presentation comparable to our study. They observed dyspnea in 77.1-84.1% of the patients with PE, pleuritic chest pain in 39.4-44.1%, non-pleuritic (substernal) in 15.2-16.3%, syncope in 5.5-26.2%, and hemoptysis in 4.5-7.6% 13-15. Slightly lower incidence of pleuritic and higher non-pleuritic chest pain (higher non-pleuritic to pleuritic ratio) observed in this cohort might be related to the cardiac-referral nature of our center. A meta-analysis reported precordial T wave inversions, S1Q3T3, and right bundle branch block (RBBB) prevalence at 29%, 24%, and 12%, respectively 16. These numbers were 19.0%, 34.8%, and 14.5% in our study, respectively. This comparison shows an increased incidence of S1Q3T3, lower observation of precordial T wave inversions, and similar RBBB rates.

Alike this cohort, hypertension, obesity, recent hospitalization, active malignancy, and smoking were the most common risk factors and underlying conditions in the EMPEROR cohort. However, they observed a much higher rate of underlying malignancy compared to us (22.3% vs. 5.7%) 13. The observed in-hospital and overall mortality rates in our study were lower than the previous studies. A study from Denmark estimated the 30-day mortality risk ranging from 17% in 2004 to 11% in 2014 17. In 2006, a year before the start of our registry, the in-hospital mortality rate in the United States was recorded at 7.8% 18. In-hospital fatality rate in Germany also ranged from 20.4% in 2005 to 13.9% in 2015 19. A study from England between 1997-2015 estimated 1- and 6-months survival rate at 85% and 72.4%, respectively 20. The multi-center international Registro Informatizado de la Enfermedad TromboEmbolica venosa (RIETE) registry reported an 8.65% 3-months mortality rate 21.

A considerable proportion of the patients in the delayed diagnosis group (18.9%) underwent coronary angiography. As several adverse events might complicate coronary angiography 22, delayed diagnosis may expose the patients to the unnecessary complications this procedure. 

This cohort suggests that a delayed PE diagnosis does not increase the risk of patients’ mortality at follow-up. This finding confirms some of the earlier studies 11, 12, 23, 24. We hypothesize that similar mortality rates relate to two phenomena. First, the patients presenting later than seven days could have less severely symptomatic forms of PE, evident in the different hemodynamic instability rates. Second, under-treatment will possibly not affect the anticoagulation therapy of the patients with delayed diagnosis, as almost all of the patients admitted to our hospital will receive anticoagulation therapy, as warranted by the acute coronary syndrome (ACS) protocol. Nevertheless, miss-recognition of their condition might expose them to over-treatment with medications highly prescribed in our center, such as aspirin and clopidogrel.

A computerized model suggested that preemptive anticoagulant therapy before PE confirmation could improve the outcomes in patients with moderate and high-risk features 25. Although we did not directly test this hypothesis, our data indirectly suggest that pre-confirmation anticoagulant therapy might positively affect the outcome of the patients with PE. We identified hemoptysis and hemodynamic instability as the parameters related to a reduced risk of delayed PE diagnosis. 

Researchers i n two earlier studies confirmed the effect of hemoptysis and expressed their surprise at this finding 5, 11. Nevertheless, most patients consider hemoptysis a serious symptom requiring urgent medical care 26, 27. Therefore, we believed they tend to admit earlier and get a PE diagnosis. Earlier research promotes this symptom as an important determinant of pretest probability 28, 29. Furthermore, as a cardiology care provider, PE puts itself at the top of our differential diagnoses list. All these points support the observed effect of hemoptysis on earlier PE diagnosis.

Hemodynamic instability at presentation indicates a critical red flag warranting an immediate diagnosis following stabilization 30. It can also serve as a root cause of conditions such as syncope that might increase the likelihood of PE and its earlier diagnosis 31, 32. We report diabetes mellitus and heart failure as indicators of increased risk of delayed diagnosis in the multivariate analysis. Earlier studies reported the presence of several comorbidities as risk factors for delayed diagnosis, such as chronic obstructive pulmonary disease (COPD), asthma, and heart failure 5, 11, 32-34. We think that these conditions might complicate the clinical picture of PE and mislead the physicians towards a more straightforward diagnosis. Furthermore, some underlying conditions, such as lung diseases, might imitate the observed PE symptoms and postpone the diagnosis 35.

This study reports the existence of inverted T waves in the precordial leads as an independent factor foretelling the risk of delayed diagnosis. This pattern presents RV dysfunction in the settings of PE 36. However, physicians can easily confuse this diagnosis with the presence of ischemia or several other cardiovascular disorders, causing this electrocardiographic pattern 37. Especially in our center, PE might be sacrificed for the sake of these cardiologic conditions.

Some earlier studies proposed the protective effect of chest pain against delayed PE diagnosis 5, 7, 38, 39. However, we did not observe any effect attributed to chest pain related to the time of diagnosis. A possible justification is that the patients who do not present with chest pain might suffer from other symptoms and risk factors (e.g., hemodynamic instability, hemoptysis, or history of recent surgery) that navigate the physicians towards the correct diagnosis. 

Well-known risk factors of PE, including previous deep vein thrombosis (D VT), recent major surgery, and recent immobilization, correlated with an earlier diagnosis 5, 7, 12, 40. Surprisingly, we did not observe such a correlation. Many patients who performed major non-cardiac surgeries in other centers seek medical care in their index surgery hospital when an acute syndrome happens. However, our center does not perform any non-cardiac surgeries. Furthermore, our emergency room protocols prioritize a timely diagnosis of major chest syndromes, such as acute coronary syndromes, PE, and aortic dissection. 

Therefore, acute PE gets special diagnostic attention, sometimes to an excessive extent. These factors might explain the lack of difference in the diagnosis of patients with and without risk factors. 

Although RV diameter significantly increased in the delayed diagnosis group at follow-up, they demonstrated no increase in moderate to severe RV dysfunction. This conclusion is in line with a previous study 23. Our explanation for this finding sums up in three factors, i.e., 1) RV dysfunction facilitates PE diagnosis and patients with normal RV function might face a delayed diagnosis; 2) delayed diagnosis group also received anticoagulation therapy for other indications, mostly ACS; and 3) patients who presented later might have lower disease severity.

Our study’s main limitation lies in its single-center nature. We believe that by covering a period of 11 years and including high-quality data on a large set of patients in our registry, we managed to overcome this concern to great extents. Additionally, our center serves as a specialized referral center, and a subset of our findings might be inapplicable for general centers. Also, the possible PE patients who were sent home with a wrong diagnosis could not be included in our study, and such events might affect the findings reported in our study.

In conclusions Hemoptysis and hemodynamic instability inversely correlated with a delayed diagnosis in the multivariate analysis. Diabetes mellitus, heart failure, and T wave inversions in precordial leads were the independent predictors of an increased risk of delayed diagnosis. Delayed PE diagnosis did not increase the patients' mortality rates. Further studies with larger populations will enlighten future perspectives of researchers and health-care workers.

### Ethical statements:

This study was approved by the local institutional review board and complies with the Declaration of Helsinki. All the participants had consent to participate.
